# Hexakis(acetonitrile-κ*N*)ruthenium(II) bis­(hexa­bromo­carbadodeca­borate) aceto­nitrile solvate

**DOI:** 10.1107/S1600536810006252

**Published:** 2010-02-24

**Authors:** Joshua Masland, Jason Diaz, Shawn Eady, Emil Lobkovsky, Anna Larsen

**Affiliations:** a Department of Chemistry, Ithaca College, 953 Danby Road, Ithaca, NY 14850, USA; b X-Ray Diffraction Facility, 167 Spencer T. Olin Laboratory, Cornell University, Ithaca, NY 14850, USA

## Abstract

The title compound, [Ru(NCCH_3_)_6_](CH_6_B_11_Br_6_)_2_·CH_3_CN, consists of the ’naked’ ruthenium(II) cation surrounded by six acetonitrile mol­ecules, each coordinated *via* the nitro­gen atoms in a linear or nearly-linear fashion in a typical octa­hedral over-all arrangement. The cation is balanced by the two hexa-bromo­carborane cage anionic fragments [CB_11_H_6_Br_6_]. Weak C—H⋯Br and B—H⋯Br inter­actions link neighboring anions.

## Related literature

For related literature pertaining to ruthenium and ruthenium derivative structures, see: Bergman & Chang (1987 ▶); Burns & Hubbard (1994 ▶); Stasko *et al.* (2002 ▶); Brookhart *et al.* (1992 ▶). For related ruthenium structures, see: Pearsal *et al.* (2007 ▶). 
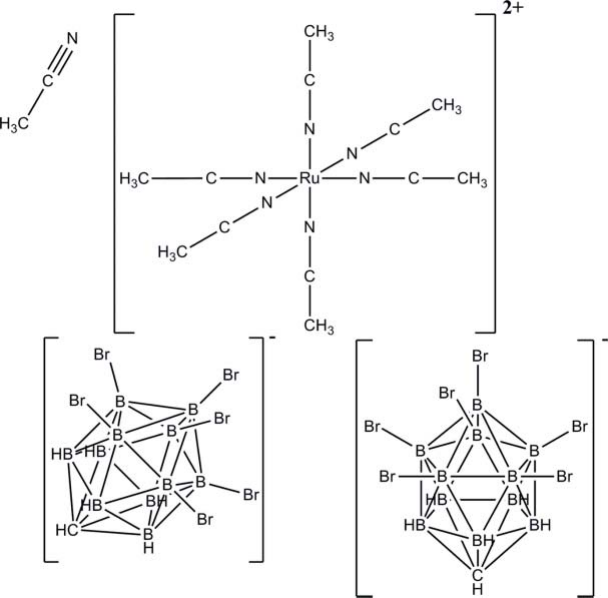


## Experimental

### 

#### Crystal data

[Ru(C_2_H_3_N)_6_](CH_6_B_11_Br_6_)_2_·C_2_H_3_N
*M* *_r_* = 1621.30
Orthorhombic, 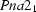

*a* = 21.332 (2) Å
*b* = 11.7577 (10) Å
*c* = 20.2620 (17) Å
*V* = 5082.1 (8) Å^3^
*Z* = 4
Mo *K*α radiation
μ = 9.77 mm^−1^
*T* = 173 K
0.20 × 0.15 × 0.10 mm

#### Data collection

Bruker CCD-1000 area-detector diffractometer
Absorption correction: multi-scan (*SADABS*; Bruker, 2007 ▶) *T*_min_ = 0.245, *T*_max_ = 0.442
36328 measured reflections
13481 independent reflections
10985 reflections with *I* > 2σ(*I*)
*R*_int_ = 0.042

#### Refinement

*R*[*F* ^2^ > 2σ(*F* ^2^)] = 0.033
*wR*(*F* ^2^) = 0.062
*S* = 0.97
13481 reflections
531 parameters
1 restraint
H-atom parameters constrained
Δρ_max_ = 0.83 e Å^−3^
Δρ_min_ = −0.73 e Å^−3^
Absolute structure: Flack (1983 ▶), 6319 Friedel pairs
Flack parameter: 0.000 (5)

### 

Data collection: *SMART* (Bruker, 2000 ▶); cell refinement: *SAINT* (Bruker, 2007 ▶); data reduction: *SAINT*; program(s) used to solve structure: *SHELXTL* (Sheldrick, 2008 ▶); program(s) used to refine structure: *SHELXTL*; molecular graphics: *SHELXTL*; software used to prepare material for publication: *SHELXTL* and *publCIF* (Westrip, 2010 ▶).

## Supplementary Material

Crystal structure: contains datablocks global, I. DOI: 10.1107/S1600536810006252/ez2197sup1.cif

Structure factors: contains datablocks I. DOI: 10.1107/S1600536810006252/ez2197Isup2.hkl

Additional supplementary materials:  crystallographic information; 3D view; checkCIF report

## Figures and Tables

**Table 1:** Hydrogen-bond geometry (Å, °)

*D*—H⋯*A*	*D*—H	H⋯*A*	*D*⋯*A*	*D*—H⋯*A*
B10—H10⋯Br8^i^	1.12	2.85	3.612 (5)	125
C1*AA*—H1*A*⋯Br4^ii^	1.12	2.77	3.547 (5)	126

Symmetry codes: (i) 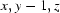; (ii) 
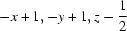
.

**Fig. 1.**
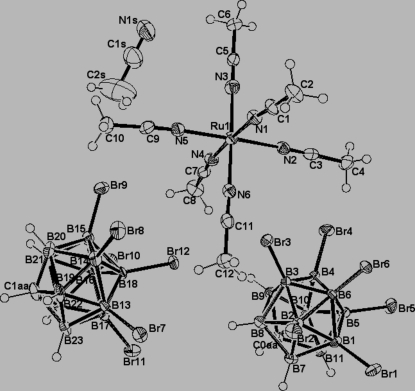
Molecular structure of (I)with atom numbering scheme. The thermal ellipsoids are shown at 50% probability level.

## supplementary crystallographic information

### Comment

Electrophilic complexes with the [Cp*Ru(NO)] core are reactive towards small molecular nucleophiles. In the presence of labile neutral ligands or weakly coordinating anions, such as trifluoromethanesulfonate, these complexes exhibit pro-catalytic reactivity with unsaturated hydrocarbons and alcohols (Burns and Hubbard, 1994; Pearsal *et al.*, 2007). The present study's goal is introduction of the non-coordinating carborane cage anions of the [CB_11_H_12_] family in order to increase the reactivity of the ruthenium catalytic center (Stasko *et al.*, 2002). The synthetic route to the desired complexes includes protonation of the dialkyl starting material with the solvated proton salt of the weakly-coordinating anion (similar to Brookhart, *et al.*, 1992). This process eventually results in stripping all the ligands off the ruthenium center to give the title compound comprised of the 'naked' hexa-acetonitrile ruthenium cationic fragment balanced by two hexa-bromo-carborane anionic fragments. The catalytic activity of this complex is currently under investigation.

### Experimental

The compound was obtained by a prolonged exposure of the Cp*Ru(NO)(CH_3_)_2_ complex to an excess of carborane-based protonating agent [(C_2_H_5_OC_2_H_5_)_2_ H]^+^ [CB_11_H_6_Br_6_]^-^ in acetonitrile.

All synthetic procedures were carried out in inert atmosphere and in anhydrous solvents. The protonating agent [(C_2_H_5_OC_2_H_5_)_2_ H]^+^ [CB_11_H_6_Br_6_]^-^ and starting ruthenium complex Cp*Ru(NO)(CH_3_)_2_ were synthesized according to the reported procedures (Stasko *et al.*, 2002; Bergman & Chang,1987).

20 mg (0.065 mmol) of Cp*Ru(NO)(CH_3_)_2_ were dissolved in 10 ml of CH_3_CN and the solution was added to 200 mg of solid [(C_2_H_5_OC_2_H_5_)_2_ H]^+^ [CB_11_H_6_Br_6_]^-^ (0.265 mmol). Vigorous evolution of a gas (methane) was observed. The color of the solution gradually changed from dark red to dark purple-red. Initial product of the reaction, [Cp*Ru(NO)(CH_3_)(NCCH_3_)] [CB_11_H_6_Br_6_], formed *via* a mono-protonation process and loss of one methane molecule from the starting material, was observed spectroscopically (by 1H NMR) in the aliquot of the reaction mixture taken after 4 hrs. The red crystals of the [Ru(NCCH_3_)_6_]] [CB_11_H_6_Br_6_]_2_ were grown from the reaction mixture in acetonitrile at ambient temperature under nitrogen by slow evaporation over a period of 3 weeks.

### Refinement

All H-atoms were placed in idealized locations and refined as riding with appropriate thermal displacement coefficients *U*_iso_(H) = 1.2 or 1.5 times *U*_eq_(bearing atom).

All H-atoms were placed in idealized locations with C—H distances of 0.981 Å for methyl carbons, and B—H and other C—H distances of 1.212 Å and refined as riding with thermal displacement coefficients *U*_iso_(H) set to 1.5 times *U*_eq_(bearing C atom) for the methyl atoms and 1.2 times *U*_eq_(bearing atom) otherwise.

### Crystal data

**Table:**

[Ru(C_2_H_3_N)_6_](CH_6_B_11_Br_6_)_2_·C_2_H_3_N	*F*(000) = 3008
*M**_r_* = 1621.30	*D*_x_ = 2.119 Mg m^−^^3^
Orthorhombic, *P**n**a*2_1_	Mo *K*α radiation, λ = 0.71073 Å
Hall symbol: P 2c -2n	Cell parameters from 999 reflections
*a* = 21.332 (2) Å	θ = 1.9–29.3°
*b* = 11.7577 (10) Å	µ = 9.77 mm^−^^1^
*c* = 20.2620 (17) Å	*T* = 173 K
*V* = 5082.1 (8) Å^3^	Block, red
*Z* = 4	0.20 × 0.15 × 0.10 mm

### Data collection

**Table:**

Bruker CCD-1000 area-detector diffractometer	13481 independent reflections
Radiation source: fine-focus sealed tube	10985 reflections with *I* > 2σ(*I*)
graphite	*R*_int_ = 0.042
0.30° ω and 0.4 ° φ scans	θ_max_ = 29.3°, θ_min_ = 1.9°
Absorption correction: multi-scan (*SADABS*; Bruker, 2007)	*h* = −29→28
*T*_min_ = 0.245, *T*_max_ = 0.442	*k* = −16→15
36328 measured reflections	*l* = −27→27

### Refinement

**Table:**

Refinement on *F*^2^	Secondary atom site location: difference Fourier map
Least-squares matrix: full	Hydrogen site location: inferred from neighbouring sites
*R*[*F*^2^ > 2σ(*F*^2^)] = 0.033	H-atom parameters constrained
*wR*(*F*^2^) = 0.062	*w* = 1/[σ^2^(*F*_o_^2^) + (0.002*P*)^2^] where *P* = (*F*_o_^2^ + 2*F*_c_^2^)/3
*S* = 0.97	(Δ/σ)_max_ = 0.001
13481 reflections	Δρ_max_ = 0.83 e Å^−^^3^
531 parameters	Δρ_min_ = −0.72 e Å^−^^3^
1 restraint	Absolute structure: Flack (1983), 6319 Friedel pairs
Primary atom site location: structure-invariant direct methods	Flack parameter: 0.000 (5)

### Special details

**Table:**

Geometry. All e.s.d.'s (except the e.s.d. in the dihedral angle between two l.s. planes) are estimated using the full covariance matrix. The cell e.s.d.'s are taken into account individually in the estimation of e.s.d.'s in distances, angles and torsion angles; correlations between e.s.d.'s in cell parameters are only used when they are defined by crystal symmetry. An approximate (isotropic) treatment of cell e.s.d.'s is used for estimating e.s.d.'s involving l.s. planes.
Refinement. Refinement of *F*^2^ against ALL reflections. The weighted *R*-factor *wR* and goodness of fit *S* are based on *F*^2^, conventional *R*-factors *R* are based on *F*, with *F* set to zero for negative *F*^2^. The threshold expression of *F*^2^ > σ(*F*^2^) is used only for calculating *R*-factors(gt) *etc*. and is not relevant to the choice of reflections for refinement. *R*-factors based on *F*^2^ are statistically about twice as large as those based on *F*, and *R*- factors based on ALL data will be even larger.

### Fractional atomic coordinates and isotropic or equivalent isotropic displacement parameters (Å^2^)

**Table:**

	*x*	*y*	*z*	*U*_iso_*/*U*_eq_	
Ru1	0.399141 (18)	0.77943 (3)	0.705666 (18)	0.02190 (8)	
N1	0.3418 (2)	0.8915 (3)	0.75141 (19)	0.0252 (9)	
N2	0.3486 (2)	0.6518 (3)	0.74612 (18)	0.0232 (9)	
N3	0.4578 (2)	0.7767 (3)	0.7844 (2)	0.0289 (9)	
N4	0.4530 (2)	0.6611 (3)	0.66043 (19)	0.0245 (9)	
N5	0.4493 (2)	0.9056 (3)	0.66337 (19)	0.0261 (9)	
N6	0.34097 (19)	0.7832 (3)	0.62615 (19)	0.0229 (8)	
C1	0.3052 (3)	0.9451 (4)	0.7782 (2)	0.0264 (11)	
C2	0.2595 (3)	1.0150 (4)	0.8113 (3)	0.0371 (13)	
H2C	0.2802	1.0603	0.8455	0.056*	
H2B	0.2276	0.9663	0.8317	0.056*	
H2A	0.2395	1.0659	0.7793	0.056*	
C3	0.3216 (2)	0.5780 (4)	0.7671 (2)	0.0271 (11)	
C4	0.2864 (3)	0.4804 (4)	0.7934 (3)	0.0355 (13)	
H4C	0.3127	0.4390	0.8250	0.053*	
H4A	0.2747	0.4297	0.7570	0.053*	
H4B	0.2485	0.5076	0.8156	0.053*	
C5	0.4903 (2)	0.7721 (4)	0.8281 (2)	0.0256 (10)	
C6	0.5326 (3)	0.7672 (4)	0.8846 (2)	0.0354 (12)	
H6B	0.5756	0.7821	0.8699	0.053*	
H6A	0.5304	0.6915	0.9047	0.053*	
H6C	0.5202	0.8246	0.9171	0.053*	
C7	0.4787 (2)	0.5888 (4)	0.6358 (2)	0.0261 (10)	
C8	0.5105 (3)	0.4935 (4)	0.6042 (3)	0.0370 (13)	
H8C	0.5355	0.4524	0.6370	0.055*	
H8B	0.5382	0.5222	0.5693	0.055*	
H8A	0.4794	0.4420	0.5849	0.055*	
C9	0.4757 (2)	0.9733 (4)	0.6354 (3)	0.0299 (11)	
C10	0.5104 (3)	1.0596 (4)	0.5984 (3)	0.0435 (15)	
H10B	0.4837	1.1264	0.5917	0.065*	
H10A	0.5229	1.0285	0.5555	0.065*	
H10C	0.5479	1.0817	0.6233	0.065*	
C11	0.3105 (2)	0.7833 (4)	0.5804 (2)	0.0256 (11)	
C12	0.2710 (3)	0.7818 (4)	0.5225 (2)	0.0324 (12)	
H12A	0.2539	0.7052	0.5163	0.049*	
H12B	0.2958	0.8032	0.4838	0.049*	
H12C	0.2365	0.8360	0.5282	0.049*	
Br1	0.09098 (2)	0.22557 (4)	0.51235 (3)	0.02750 (11)	
Br2	0.17891 (3)	0.50024 (3)	0.48909 (2)	0.02850 (11)	
Br3	0.32771 (2)	0.48584 (3)	0.59669 (2)	0.02548 (10)	
Br4	0.33173 (3)	0.19992 (4)	0.68133 (3)	0.03209 (12)	
Br5	0.18567 (3)	0.03424 (4)	0.63167 (3)	0.03231 (12)	
Br6	0.17687 (2)	0.35779 (4)	0.65791 (2)	0.02565 (10)	
C0AA	0.2963 (2)	0.1822 (4)	0.4613 (2)	0.0220 (10)	
H0A	0.3221	0.1438	0.4186	0.026*	
B1	0.1813 (2)	0.2282 (4)	0.5161 (3)	0.0195 (10)	
B2	0.2239 (2)	0.3581 (4)	0.5052 (2)	0.0191 (10)	
B3	0.2923 (2)	0.3521 (4)	0.5546 (2)	0.0180 (10)	
B4	0.2940 (2)	0.2181 (4)	0.5941 (3)	0.0197 (10)	
B5	0.2248 (3)	0.1416 (4)	0.5710 (2)	0.0188 (10)	
B6	0.2212 (2)	0.2911 (4)	0.5838 (2)	0.0147 (10)	
B7	0.2284 (3)	0.2502 (4)	0.4441 (3)	0.0229 (12)	
H7	0.2077	0.2598	0.3937	0.028*	
B8	0.2965 (3)	0.3270 (4)	0.4675 (3)	0.0233 (11)	
H8	0.3204	0.3869	0.4327	0.028*	
B9	0.3402 (3)	0.2401 (4)	0.5234 (3)	0.0225 (11)	
H9	0.3926	0.2433	0.5250	0.027*	
B10	0.2980 (3)	0.1107 (4)	0.5337 (3)	0.0232 (11)	
H10	0.3232	0.0283	0.5422	0.028*	
B11	0.2287 (3)	0.1170 (4)	0.4860 (3)	0.0218 (11)	
H11	0.2080	0.0389	0.4630	0.026*	
Br7	0.32987 (2)	0.77287 (4)	0.33363 (2)	0.02870 (11)	
Br8	0.42000 (3)	0.93866 (4)	0.46079 (3)	0.03373 (12)	
Br9	0.56818 (3)	0.76830 (4)	0.50431 (3)	0.03494 (12)	
Br10	0.56984 (2)	0.50141 (4)	0.39952 (3)	0.02875 (11)	
Br11	0.42319 (3)	0.49971 (4)	0.29036 (3)	0.03068 (11)	
Br12	0.41484 (2)	0.61228 (4)	0.46759 (2)	0.02423 (10)	
C1AA	0.5353 (3)	0.8272 (4)	0.2853 (2)	0.0291 (11)	
H1A	0.5610	0.8742	0.2455	0.035*	
B13	0.4202 (3)	0.7666 (4)	0.3357 (3)	0.0220 (11)	
B14	0.4631 (3)	0.8445 (4)	0.3960 (3)	0.0246 (12)	
B15	0.5318 (3)	0.7669 (4)	0.4164 (3)	0.0235 (11)	
B16	0.5325 (3)	0.6418 (4)	0.3670 (2)	0.0195 (11)	
B17	0.4641 (3)	0.6413 (4)	0.3173 (2)	0.0212 (11)	
B18	0.4606 (2)	0.6923 (4)	0.4001 (2)	0.0170 (10)	
B19	0.4671 (3)	0.8845 (4)	0.3124 (3)	0.0263 (12)	
H19	0.4458	0.9654	0.2937	0.032*	
B20	0.5362 (3)	0.8849 (5)	0.3618 (3)	0.0286 (13)	
H20	0.5607	0.9658	0.3756	0.034*	
B21	0.5796 (3)	0.7595 (4)	0.3441 (3)	0.0277 (13)	
H21	0.6321	0.7578	0.3462	0.033*	
B22	0.5369 (3)	0.6817 (4)	0.2831 (3)	0.0252 (12)	
H22	0.5615	0.6288	0.2452	0.030*	
B23	0.4681 (3)	0.7597 (5)	0.2636 (3)	0.0277 (13)	
H23	0.4479	0.7584	0.2126	0.033*	
N1S	0.6665 (3)	0.8100 (6)	0.7904 (3)	0.083 (2)	
C1S	0.6582 (4)	0.8053 (6)	0.7357 (4)	0.063 (2)	
C2S	0.6476 (6)	0.8005 (6)	0.6695 (4)	0.126 (5)	
H2SC	0.6778	0.8495	0.6466	0.189*	
H2SB	0.6050	0.8266	0.6600	0.189*	
H2SA	0.6526	0.7220	0.6542	0.189*	

### Atomic displacement parameters (Å^2^)

**Table:**

	*U*^11^	*U*^22^	*U*^33^	*U*^12^	*U*^13^	*U*^23^
Ru1	0.0257 (2)	0.01802 (17)	0.02196 (19)	−0.00100 (15)	0.00163 (16)	−0.00001 (15)
N1	0.032 (3)	0.0193 (18)	0.024 (2)	0.0028 (17)	0.0022 (19)	0.0000 (16)
N2	0.030 (2)	0.0195 (18)	0.019 (2)	0.0012 (16)	0.0004 (17)	0.0005 (16)
N3	0.033 (3)	0.0214 (19)	0.033 (2)	−0.0021 (18)	0.002 (2)	−0.0021 (18)
N4	0.030 (2)	0.0212 (19)	0.022 (2)	0.0006 (17)	−0.0014 (18)	−0.0010 (16)
N5	0.031 (2)	0.0239 (19)	0.024 (2)	−0.0030 (17)	0.0016 (19)	−0.0023 (17)
N6	0.028 (2)	0.0170 (18)	0.023 (2)	0.0010 (15)	0.0021 (18)	0.0010 (16)
C1	0.036 (3)	0.021 (2)	0.021 (3)	0.002 (2)	0.004 (2)	0.0048 (19)
C2	0.039 (4)	0.046 (3)	0.026 (3)	0.010 (3)	0.005 (2)	0.001 (2)
C3	0.028 (3)	0.031 (3)	0.022 (3)	0.002 (2)	−0.006 (2)	−0.004 (2)
C4	0.044 (4)	0.029 (3)	0.034 (3)	−0.015 (2)	−0.006 (3)	0.005 (2)
C5	0.026 (3)	0.024 (2)	0.026 (3)	−0.005 (2)	0.004 (2)	−0.002 (2)
C6	0.033 (3)	0.044 (3)	0.029 (3)	−0.011 (2)	−0.005 (2)	−0.004 (2)
C7	0.026 (3)	0.028 (2)	0.024 (3)	−0.007 (2)	−0.006 (2)	0.003 (2)
C8	0.036 (3)	0.037 (3)	0.037 (3)	0.014 (2)	0.003 (3)	−0.004 (2)
C9	0.030 (3)	0.028 (2)	0.031 (3)	−0.008 (2)	−0.002 (2)	−0.007 (2)
C10	0.057 (4)	0.042 (3)	0.032 (3)	−0.023 (3)	0.008 (3)	−0.002 (3)
C11	0.032 (3)	0.019 (2)	0.026 (3)	−0.0016 (19)	0.005 (2)	0.0027 (19)
C12	0.037 (3)	0.027 (2)	0.033 (3)	−0.001 (2)	−0.008 (2)	0.004 (2)
Br1	0.0190 (2)	0.0286 (2)	0.0349 (3)	−0.00053 (19)	−0.0065 (2)	−0.0091 (2)
Br2	0.0357 (3)	0.0201 (2)	0.0297 (3)	0.00673 (19)	−0.0053 (2)	0.00434 (19)
Br3	0.0292 (3)	0.0224 (2)	0.0248 (2)	−0.01019 (18)	−0.0001 (2)	−0.00055 (19)
Br4	0.0332 (3)	0.0362 (3)	0.0269 (3)	−0.0033 (2)	−0.0145 (2)	0.0097 (2)
Br5	0.0342 (3)	0.0264 (2)	0.0362 (3)	−0.0099 (2)	−0.0006 (2)	0.0116 (2)
Br6	0.0253 (3)	0.0345 (2)	0.0171 (2)	−0.0024 (2)	0.0058 (2)	−0.00666 (19)
C0AA	0.026 (3)	0.024 (2)	0.017 (2)	0.0020 (19)	0.002 (2)	−0.0049 (18)
B1	0.017 (2)	0.018 (2)	0.023 (3)	0.001 (2)	−0.002 (2)	−0.005 (2)
B2	0.018 (3)	0.024 (2)	0.015 (3)	−0.0007 (19)	0.000 (2)	0.001 (2)
B3	0.017 (3)	0.019 (2)	0.019 (3)	−0.0014 (19)	0.002 (2)	0.0022 (19)
B4	0.017 (2)	0.017 (2)	0.025 (3)	−0.0018 (19)	−0.002 (2)	0.001 (2)
B5	0.019 (3)	0.018 (2)	0.020 (3)	0.001 (2)	−0.003 (2)	0.0040 (19)
B6	0.019 (3)	0.015 (2)	0.010 (2)	−0.0009 (18)	0.0012 (18)	0.0004 (18)
B7	0.025 (3)	0.026 (3)	0.018 (3)	0.001 (2)	−0.003 (2)	−0.0032 (19)
B8	0.025 (3)	0.026 (3)	0.019 (3)	0.004 (2)	0.000 (2)	0.000 (2)
B9	0.023 (3)	0.024 (3)	0.021 (3)	0.004 (2)	−0.002 (2)	0.000 (2)
B10	0.023 (3)	0.020 (2)	0.027 (3)	0.003 (2)	0.001 (2)	0.000 (2)
B11	0.021 (3)	0.020 (2)	0.025 (3)	0.001 (2)	0.000 (2)	−0.006 (2)
Br7	0.0210 (3)	0.0375 (3)	0.0276 (3)	0.0067 (2)	−0.0004 (2)	0.0066 (2)
Br8	0.0399 (3)	0.0236 (2)	0.0376 (3)	0.0011 (2)	0.0123 (2)	−0.0059 (2)
Br9	0.0384 (3)	0.0375 (3)	0.0290 (3)	−0.0131 (2)	−0.0098 (2)	0.0016 (2)
Br10	0.0275 (3)	0.0234 (2)	0.0353 (3)	0.0061 (2)	−0.0020 (2)	0.00958 (19)
Br11	0.0340 (3)	0.0301 (2)	0.0279 (3)	0.0008 (2)	−0.0059 (2)	−0.0072 (2)
Br12	0.0267 (3)	0.0265 (2)	0.0195 (2)	−0.00456 (18)	0.0039 (2)	0.00747 (18)
C1AA	0.030 (3)	0.029 (2)	0.028 (3)	0.006 (2)	0.008 (2)	0.014 (2)
B13	0.024 (3)	0.030 (3)	0.012 (2)	0.003 (2)	0.004 (2)	0.004 (2)
B14	0.030 (3)	0.017 (2)	0.027 (3)	0.002 (2)	0.007 (2)	−0.002 (2)
B15	0.026 (3)	0.022 (2)	0.023 (3)	−0.003 (2)	−0.001 (2)	0.007 (2)
B16	0.022 (3)	0.023 (3)	0.013 (2)	0.004 (2)	0.002 (2)	0.011 (2)
B17	0.021 (3)	0.025 (3)	0.017 (3)	0.005 (2)	0.000 (2)	0.003 (2)
B18	0.017 (3)	0.021 (2)	0.013 (2)	−0.0002 (19)	0.002 (2)	0.0072 (19)
B19	0.024 (3)	0.024 (3)	0.032 (3)	0.005 (2)	0.007 (2)	0.017 (2)
B20	0.026 (3)	0.024 (3)	0.036 (3)	−0.006 (2)	0.009 (3)	0.002 (2)
B21	0.019 (3)	0.030 (3)	0.035 (3)	−0.002 (2)	0.007 (2)	0.010 (2)
B22	0.029 (3)	0.029 (3)	0.018 (3)	0.006 (2)	0.007 (2)	0.008 (2)
B23	0.025 (3)	0.036 (3)	0.022 (3)	0.008 (2)	0.007 (2)	0.013 (2)
N1S	0.069 (5)	0.136 (6)	0.044 (4)	−0.032 (4)	−0.001 (3)	0.016 (4)
C1S	0.067 (5)	0.067 (5)	0.053 (5)	0.000 (4)	0.000 (4)	0.016 (4)
C2S	0.236 (15)	0.059 (5)	0.083 (7)	0.032 (7)	−0.043 (8)	−0.017 (5)

### Geometric parameters (Å, °)

**Table:**

Ru1—N5	2.020 (4)	B4—B6	1.785 (7)
Ru1—N2	2.021 (4)	B4—B5	1.790 (7)
Ru1—N1	2.022 (4)	B5—B11	1.750 (7)
Ru1—N4	2.024 (4)	B5—B10	1.773 (7)
Ru1—N3	2.028 (4)	B5—B6	1.777 (6)
Ru1—N6	2.034 (4)	B7—B8	1.776 (8)
N1—C1	1.142 (6)	B7—B11	1.780 (7)
N2—C3	1.126 (6)	B7—H7	1.1200
N3—C5	1.127 (6)	B8—B9	1.786 (7)
N4—C7	1.127 (6)	B8—H8	1.1200
N5—C9	1.126 (6)	B9—B10	1.781 (7)
N6—C11	1.132 (6)	B9—H9	1.1200
C1—C2	1.441 (7)	B10—B11	1.768 (8)
C2—H2C	0.9800	B10—H10	1.1200
C2—H2B	0.9800	B11—H11	1.1200
C2—H2A	0.9800	Br7—B13	1.928 (6)
C3—C4	1.470 (7)	Br8—B14	1.948 (5)
C4—H4C	0.9800	Br9—B15	1.944 (5)
C4—H4A	0.9800	Br10—B16	1.947 (5)
C4—H4B	0.9800	Br11—B17	1.958 (5)
C5—C6	1.457 (7)	Br12—B18	1.926 (5)
C6—H6B	0.9800	C1AA—B20	1.694 (7)
C6—H6A	0.9800	C1AA—B19	1.695 (7)
C6—H6C	0.9800	C1AA—B23	1.696 (8)
C7—C8	1.459 (7)	C1AA—B22	1.712 (7)
C8—H8C	0.9800	C1AA—B21	1.717 (7)
C8—H8B	0.9800	C1AA—H1A	1.1200
C8—H8A	0.9800	B13—B19	1.774 (7)
C9—C10	1.463 (7)	B13—B14	1.780 (8)
C10—H10B	0.9800	B13—B17	1.785 (7)
C10—H10A	0.9800	B13—B23	1.787 (8)
C10—H10C	0.9800	B13—B18	1.791 (7)
C11—C12	1.445 (7)	B14—B19	1.759 (7)
C12—H12A	0.9800	B14—B20	1.773 (8)
C12—H12B	0.9800	B14—B15	1.776 (8)
C12—H12C	0.9800	B14—B18	1.792 (6)
Br1—B1	1.929 (5)	B15—B20	1.776 (7)
Br2—B2	1.955 (5)	B15—B16	1.778 (7)
Br3—B3	1.943 (5)	B15—B18	1.784 (7)
Br4—B4	1.954 (5)	B15—B21	1.786 (8)
Br5—B5	1.950 (5)	B16—B22	1.767 (7)
Br6—B6	1.940 (5)	B16—B21	1.772 (7)
C0AA—B7	1.690 (7)	B16—B17	1.773 (7)
C0AA—B10	1.693 (7)	B16—B18	1.776 (7)
C0AA—B8	1.707 (7)	B17—B22	1.765 (8)
C0AA—B11	1.707 (7)	B17—B23	1.770 (7)
C0AA—B9	1.710 (7)	B17—B18	1.782 (7)
C0AA—H0A	1.1200	B19—B23	1.770 (8)
B1—B11	1.761 (7)	B19—B20	1.782 (8)
B1—B5	1.770 (7)	B19—H19	1.1200
B1—B6	1.776 (7)	B20—B21	1.778 (8)
B1—B7	1.789 (7)	B20—H20	1.1200
B1—B2	1.790 (7)	B21—B22	1.788 (8)
B2—B8	1.765 (7)	B21—H21	1.1200
B2—B3	1.770 (7)	B22—B23	1.774 (8)
B2—B7	1.774 (7)	B22—H22	1.1200
B2—B6	1.779 (7)	B23—H23	1.1200
B3—B4	1.767 (7)	N1S—C1S	1.124 (9)
B3—B6	1.779 (7)	C1S—C2S	1.361 (10)
B3—B9	1.782 (7)	C2S—H2SC	0.9800
B3—B8	1.790 (7)	C2S—H2SB	0.9800
B4—B9	1.759 (7)	C2S—H2SA	0.9800
B4—B10	1.760 (7)		
			
N5—Ru1—N2	178.82 (15)	B4—B10—B11	108.0 (4)
N5—Ru1—N1	92.08 (16)	C0AA—B10—B5	104.4 (4)
N2—Ru1—N1	88.58 (15)	B4—B10—B5	60.9 (3)
N5—Ru1—N4	90.67 (15)	B11—B10—B5	59.2 (3)
N2—Ru1—N4	88.65 (15)	C0AA—B10—B9	58.9 (3)
N1—Ru1—N4	177.05 (16)	B4—B10—B9	59.5 (3)
N5—Ru1—N3	91.08 (16)	B11—B10—B9	108.8 (4)
N2—Ru1—N3	89.88 (16)	B5—B10—B9	108.6 (4)
N1—Ru1—N3	91.28 (16)	C0AA—B10—H10	125.0
N4—Ru1—N3	89.73 (16)	B4—B10—H10	122.5
N5—Ru1—N6	88.36 (15)	B11—B10—H10	121.4
N2—Ru1—N6	90.68 (15)	B5—B10—H10	122.3
N1—Ru1—N6	88.86 (16)	B9—B10—H10	121.0
N4—Ru1—N6	90.16 (15)	C0AA—B11—B5	104.8 (4)
N3—Ru1—N6	179.43 (17)	C0AA—B11—B1	104.7 (3)
C1—N1—Ru1	172.6 (4)	B5—B11—B1	60.6 (3)
C3—N2—Ru1	177.4 (4)	C0AA—B11—B10	58.3 (3)
C5—N3—Ru1	178.2 (4)	B5—B11—B10	60.5 (3)
C7—N4—Ru1	173.9 (4)	B1—B11—B10	108.8 (4)
C9—N5—Ru1	174.9 (4)	C0AA—B11—B7	57.9 (3)
C11—N6—Ru1	177.2 (4)	B5—B11—B7	108.8 (3)
N1—C1—C2	178.8 (5)	B1—B11—B7	60.7 (3)
C1—C2—H2C	109.5	B10—B11—B7	107.5 (4)
C1—C2—H2B	109.5	C0AA—B11—H11	125.4
H2C—C2—H2B	109.5	B5—B11—H11	121.7
C1—C2—H2A	109.5	B1—B11—H11	121.7
H2C—C2—H2A	109.5	B10—B11—H11	121.5
H2B—C2—H2A	109.5	B7—B11—H11	121.5
N2—C3—C4	178.9 (5)	B20—C1AA—B19	63.5 (3)
C3—C4—H4C	109.5	B20—C1AA—B23	115.8 (4)
C3—C4—H4A	109.5	B19—C1AA—B23	62.9 (3)
H4C—C4—H4A	109.5	B20—C1AA—B22	115.1 (4)
C3—C4—H4B	109.5	B19—C1AA—B22	115.0 (4)
H4C—C4—H4B	109.5	B23—C1AA—B22	62.8 (3)
H4A—C4—H4B	109.5	B20—C1AA—B21	62.8 (3)
N3—C5—C6	179.6 (6)	B19—C1AA—B21	115.5 (4)
C5—C6—H6B	109.5	B23—C1AA—B21	115.4 (4)
C5—C6—H6A	109.5	B22—C1AA—B21	62.8 (3)
H6B—C6—H6A	109.5	B20—C1AA—H1A	117.1
C5—C6—H6C	109.5	B19—C1AA—H1A	117.2
H6B—C6—H6C	109.5	B23—C1AA—H1A	117.2
H6A—C6—H6C	109.5	B22—C1AA—H1A	117.8
N4—C7—C8	178.6 (5)	B21—C1AA—H1A	117.3
C7—C8—H8C	109.5	B19—B13—B14	59.4 (3)
C7—C8—H8B	109.5	B19—B13—B17	107.0 (4)
H8C—C8—H8B	109.5	B14—B13—B17	107.3 (4)
C7—C8—H8A	109.5	B19—B13—B23	59.6 (3)
H8C—C8—H8A	109.5	B14—B13—B23	106.9 (4)
H8B—C8—H8A	109.5	B17—B13—B23	59.4 (3)
N5—C9—C10	179.0 (6)	B19—B13—B18	107.6 (4)
C9—C10—H10B	109.5	B14—B13—B18	60.2 (3)
C9—C10—H10A	109.5	B17—B13—B18	59.8 (3)
H10B—C10—H10A	109.5	B23—B13—B18	107.4 (4)
C9—C10—H10C	109.5	B19—B13—Br7	121.9 (3)
H10B—C10—H10C	109.5	B14—B13—Br7	120.6 (3)
H10A—C10—H10C	109.5	B17—B13—Br7	123.5 (3)
N6—C11—C12	179.0 (5)	B23—B13—Br7	123.8 (4)
C11—C12—H12A	109.5	B18—B13—Br7	121.1 (3)
C11—C12—H12B	109.5	B19—B14—B20	60.6 (3)
H12A—C12—H12B	109.5	B19—B14—B15	108.7 (4)
C11—C12—H12C	109.5	B20—B14—B15	60.1 (3)
H12A—C12—H12C	109.5	B19—B14—B13	60.2 (3)
H12B—C12—H12C	109.5	B20—B14—B13	108.8 (4)
B7—C0AA—B10	115.6 (4)	B15—B14—B13	108.6 (3)
B7—C0AA—B8	63.0 (3)	B19—B14—B18	108.2 (4)
B10—C0AA—B8	115.5 (3)	B20—B14—B18	108.1 (4)
B7—C0AA—B11	63.2 (3)	B15—B14—B18	60.0 (3)
B10—C0AA—B11	62.7 (3)	B13—B14—B18	60.2 (3)
B8—C0AA—B11	115.4 (4)	B19—B14—Br8	121.4 (3)
B7—C0AA—B9	115.6 (3)	B20—B14—Br8	121.8 (3)
B10—C0AA—B9	63.1 (3)	B15—B14—Br8	121.6 (4)
B8—C0AA—B9	63.0 (3)	B13—B14—Br8	120.9 (4)
B11—C0AA—B9	115.2 (4)	B18—B14—Br8	121.5 (3)
B7—C0AA—H0A	117.0	B14—B15—B20	59.9 (3)
B10—C0AA—H0A	117.3	B14—B15—B16	107.6 (4)
B8—C0AA—H0A	117.3	B20—B15—B16	107.2 (4)
B11—C0AA—H0A	117.5	B14—B15—B18	60.5 (3)
B9—C0AA—H0A	117.3	B20—B15—B18	108.3 (4)
B11—B1—B5	59.4 (3)	B16—B15—B18	59.8 (3)
B11—B1—B6	107.6 (4)	B14—B15—B21	107.8 (4)
B5—B1—B6	60.2 (3)	B20—B15—B21	59.9 (3)
B11—B1—B7	60.2 (3)	B16—B15—B21	59.6 (3)
B5—B1—B7	107.6 (4)	B18—B15—B21	108.1 (4)
B6—B1—B7	107.5 (3)	B14—B15—Br9	122.6 (3)
B11—B1—B2	107.4 (4)	B20—B15—Br9	122.8 (3)
B5—B1—B2	107.6 (4)	B16—B15—Br9	121.2 (3)
B6—B1—B2	59.8 (3)	B18—B15—Br9	120.9 (3)
B7—B1—B2	59.4 (3)	B21—B15—Br9	121.6 (4)
B11—B1—Br1	123.2 (3)	B22—B16—B21	60.7 (3)
B5—B1—Br1	122.6 (3)	B22—B16—B17	59.8 (3)
B6—B1—Br1	121.0 (3)	B21—B16—B17	108.7 (4)
B7—B1—Br1	122.1 (3)	B22—B16—B18	108.6 (4)
B2—B1—Br1	121.0 (3)	B21—B16—B18	109.1 (4)
B8—B2—B3	60.8 (3)	B17—B16—B18	60.3 (3)
B8—B2—B7	60.2 (3)	B22—B16—B15	108.8 (3)
B3—B2—B7	108.7 (4)	B21—B16—B15	60.4 (3)
B8—B2—B6	108.9 (4)	B17—B16—B15	108.3 (4)
B3—B2—B6	60.2 (3)	B18—B16—B15	60.3 (3)
B7—B2—B6	108.0 (3)	B22—B16—Br10	121.9 (3)
B8—B2—B1	108.8 (3)	B21—B16—Br10	121.3 (3)
B3—B2—B1	108.3 (3)	B17—B16—Br10	121.7 (3)
B7—B2—B1	60.2 (3)	B18—B16—Br10	120.6 (3)
B6—B2—B1	59.7 (3)	B15—B16—Br10	121.0 (3)
B8—B2—Br2	122.4 (3)	B22—B17—B23	60.3 (3)
B3—B2—Br2	122.2 (3)	B22—B17—B16	59.9 (3)
B7—B2—Br2	121.5 (3)	B23—B17—B16	107.9 (4)
B6—B2—Br2	120.8 (3)	B22—B17—B18	108.4 (4)
B1—B2—Br2	120.1 (3)	B23—B17—B18	108.5 (4)
B4—B3—B2	108.0 (4)	B16—B17—B18	59.9 (3)
B4—B3—B6	60.5 (3)	B22—B17—B13	108.8 (4)
B2—B3—B6	60.2 (3)	B23—B17—B13	60.3 (3)
B4—B3—B9	59.4 (3)	B16—B17—B13	108.1 (4)
B2—B3—B9	107.6 (4)	B18—B17—B13	60.3 (3)
B6—B3—B9	108.0 (3)	B22—B17—Br11	120.7 (3)
B4—B3—B8	107.4 (3)	B23—B17—Br11	121.2 (3)
B2—B3—B8	59.4 (3)	B16—B17—Br11	121.9 (3)
B6—B3—B8	107.8 (4)	B18—B17—Br11	122.0 (3)
B9—B3—B8	60.0 (3)	B13—B17—Br11	121.7 (4)
B4—B3—Br3	120.9 (3)	B16—B18—B17	59.8 (3)
B2—B3—Br3	122.5 (3)	B16—B18—B15	59.9 (3)
B6—B3—Br3	120.8 (3)	B17—B18—B15	107.7 (4)
B9—B3—Br3	122.0 (3)	B16—B18—B13	107.7 (3)
B8—B3—Br3	123.1 (3)	B17—B18—B13	59.9 (3)
B9—B4—B10	60.8 (3)	B15—B18—B13	107.7 (3)
B9—B4—B3	60.7 (3)	B16—B18—B14	106.9 (4)
B10—B4—B3	109.0 (4)	B17—B18—B14	106.9 (3)
B9—B4—B6	108.8 (3)	B15—B18—B14	59.6 (3)
B10—B4—B6	107.9 (4)	B13—B18—B14	59.5 (3)
B3—B4—B6	60.1 (3)	B16—B18—Br12	122.8 (3)
B9—B4—B5	108.9 (4)	B17—B18—Br12	121.6 (3)
B10—B4—B5	59.9 (3)	B15—B18—Br12	122.7 (3)
B3—B4—B5	108.2 (4)	B13—B18—Br12	120.7 (3)
B6—B4—B5	59.6 (3)	B14—B18—Br12	122.4 (3)
B9—B4—Br4	121.5 (3)	C1AA—B19—B14	104.4 (4)
B10—B4—Br4	122.0 (3)	C1AA—B19—B23	58.6 (3)
B3—B4—Br4	121.1 (3)	B14—B19—B23	108.5 (4)
B6—B4—Br4	121.1 (3)	C1AA—B19—B13	105.1 (4)
B5—B4—Br4	121.4 (3)	B14—B19—B13	60.5 (3)
B11—B5—B1	60.0 (3)	B23—B19—B13	60.6 (3)
B11—B5—B10	60.3 (3)	C1AA—B19—B20	58.2 (3)
B1—B5—B10	108.2 (4)	B14—B19—B20	60.1 (3)
B11—B5—B6	108.0 (3)	B23—B19—B20	107.9 (4)
B1—B5—B6	60.1 (3)	B13—B19—B20	108.6 (4)
B10—B5—B6	107.7 (4)	C1AA—B19—H19	125.1
B11—B5—B4	107.5 (4)	B14—B19—H19	122.2
B1—B5—B4	107.9 (3)	B23—B19—H19	121.3
B10—B5—B4	59.2 (3)	B13—B19—H19	121.7
B6—B5—B4	60.1 (3)	B20—B19—H19	121.5
B11—B5—Br5	122.3 (3)	C1AA—B20—B14	103.9 (4)
B1—B5—Br5	123.0 (3)	C1AA—B20—B15	104.8 (4)
B10—B5—Br5	120.8 (3)	B14—B20—B15	60.1 (3)
B6—B5—Br5	122.0 (3)	C1AA—B20—B21	59.2 (3)
B4—B5—Br5	120.9 (3)	B14—B20—B21	108.4 (4)
B1—B6—B5	59.8 (3)	B15—B20—B21	60.4 (3)
B1—B6—B2	60.5 (3)	C1AA—B20—B19	58.3 (3)
B5—B6—B2	107.8 (3)	B14—B20—B19	59.3 (3)
B1—B6—B3	108.6 (3)	B15—B20—B19	107.6 (4)
B5—B6—B3	108.2 (3)	B21—B20—B19	108.3 (4)
B2—B6—B3	59.7 (3)	C1AA—B20—H20	125.0
B1—B6—B4	107.8 (3)	B14—B20—H20	122.8
B5—B6—B4	60.3 (3)	B15—B20—H20	122.3
B2—B6—B4	106.8 (3)	B21—B20—H20	120.8
B3—B6—B4	59.4 (3)	B19—B20—H20	121.8
B1—B6—Br6	122.2 (3)	C1AA—B21—B16	103.4 (4)
B5—B6—Br6	122.3 (3)	C1AA—B21—B20	57.9 (3)
B2—B6—Br6	122.0 (3)	B16—B21—B20	107.4 (4)
B3—B6—Br6	120.7 (3)	C1AA—B21—B15	103.4 (4)
B4—B6—Br6	121.9 (3)	B16—B21—B15	60.0 (3)
C0AA—B7—B2	104.0 (4)	B20—B21—B15	59.8 (3)
C0AA—B7—B8	59.0 (3)	C1AA—B21—B22	58.4 (3)
B2—B7—B8	59.6 (3)	B16—B21—B22	59.5 (3)
C0AA—B7—B11	58.9 (3)	B20—B21—B22	107.4 (4)
B2—B7—B11	107.3 (3)	B15—B21—B22	107.5 (4)
B8—B7—B11	108.5 (4)	C1AA—B21—H21	125.7
C0AA—B7—B1	104.2 (4)	B16—B21—H21	122.8
B2—B7—B1	60.3 (3)	B20—B21—H21	121.8
B8—B7—B1	108.4 (4)	B15—B21—H21	122.7
B11—B7—B1	59.1 (3)	B22—B21—H21	121.8
C0AA—B7—H7	125.0	C1AA—B22—B17	104.0 (4)
B2—B7—H7	122.8	C1AA—B22—B16	103.9 (4)
B8—B7—H7	121.0	B17—B22—B16	60.3 (3)
B11—B7—H7	121.7	C1AA—B22—B23	58.2 (3)
B1—B7—H7	122.5	B17—B22—B23	60.0 (3)
C0AA—B8—B2	103.7 (4)	B16—B22—B23	108.0 (4)
C0AA—B8—B7	58.0 (3)	C1AA—B22—B21	58.7 (3)
B2—B8—B7	60.1 (3)	B17—B22—B21	108.3 (4)
C0AA—B8—B9	58.6 (3)	B16—B22—B21	59.8 (3)
B2—B8—B9	107.6 (4)	B23—B22—B21	108.1 (4)
B7—B8—B9	107.8 (4)	C1AA—B22—H22	125.5
C0AA—B8—B3	103.8 (3)	B17—B22—H22	122.3
B2—B8—B3	59.7 (3)	B16—B22—H22	122.6
B7—B8—B3	107.8 (4)	B23—B22—H22	121.5
B9—B8—B3	59.8 (3)	B21—B22—H22	121.2
C0AA—B8—H8	125.6	C1AA—B23—B19	58.5 (3)
B2—B8—H8	122.7	C1AA—B23—B17	104.4 (4)
B7—B8—H8	121.5	B19—B23—B17	107.9 (4)
B9—B8—H8	121.5	C1AA—B23—B22	59.1 (3)
B3—B8—H8	122.7	B19—B23—B22	108.3 (4)
C0AA—B9—B4	103.5 (4)	B17—B23—B22	59.7 (3)
C0AA—B9—B10	58.0 (3)	C1AA—B23—B13	104.5 (4)
B4—B9—B10	59.7 (3)	B19—B23—B13	59.8 (3)
C0AA—B9—B3	103.9 (4)	B17—B23—B13	60.2 (3)
B4—B9—B3	59.9 (3)	B22—B23—B13	108.3 (4)
B10—B9—B3	107.4 (4)	C1AA—B23—H23	124.9
C0AA—B9—B8	58.4 (3)	B19—B23—H23	121.5
B4—B9—B8	107.9 (4)	B17—B23—H23	122.6
B10—B9—B8	107.5 (4)	B22—B23—H23	121.2
B3—B9—B8	60.2 (3)	B13—B23—H23	122.3
C0AA—B9—H9	125.6	N1S—C1S—C2S	179.4 (10)
B4—B9—H9	122.7	C1S—C2S—H2SC	109.5
B10—B9—H9	122.0	C1S—C2S—H2SB	109.5
B3—B9—H9	122.5	H2SC—C2S—H2SB	109.5
B8—B9—H9	121.4	C1S—C2S—H2SA	109.5
C0AA—B10—B4	104.2 (3)	H2SC—C2S—H2SA	109.5
C0AA—B10—B11	59.1 (3)	H2SB—C2S—H2SA	109.5

### Hydrogen-bond geometry (Å, °)

**Table:**

*D*—H···*A*	*D*—H	H···*A*	*D*···*A*	*D*—H···*A*
B10—H10···Br8^i^	1.12	2.85	3.612 (5)	125
C1AA—H1A···Br4^ii^	1.12	2.77	3.547 (5)	126

Symmetry codes: (i) *x*, *y*−1, *z*; (ii) −*x*+1, −*y*+1, *z*−1/2.
